# An Instrumental High-Frequency Smart Meter with Embedded Energy Disaggregation

**DOI:** 10.3390/s25175280

**Published:** 2025-08-25

**Authors:** Dimitrios Kolosov, Matthew Robinson, Pascal A. Schirmer, Iosif Mporas

**Affiliations:** Intelligent Control Autonomous Systems Lab, School of Physics, Engineering and Computer Science, University of Hertfordshire, Hatfield AL10 9AB, UK; d.kolosov2@herts.ac.uk (D.K.); m.robinson20@herts.ac.uk (M.R.); i.mporas@herts.ac.uk (I.M.)

**Keywords:** smart meter, energy disaggregation, non-intrusive load monitoring (NILM), AI on the edge

## Abstract

Most available smart meters sample at low rates and transmit the acquired measurements to a cloud server for further processing. This article presents a prototype smart meter operating at a high sampling frequency (15 kHz) and performing energy disaggregation locally, thus negating the need to transmit the acquired high-frequency measurements. The prototype’s architecture comprises a custom signal conditioning circuit and an embedded board that performs energy disaggregation using a deep learning model. The influence of the sampling frequency on the model’s accuracy and the edge device power consumption, throughput, and latency across different hardware platforms is evaluated. The architecture embeds NILM inference into the meter hardware while maintaining a compact and energy-efficient design. The presented smart meter is benchmarked across six embedded platforms, evaluating model accuracy, latency, power usage, and throughput. Furthermore, three novel hardware-aware performance metrics are introduced to quantify NILM efficiency per unit cost, throughput, and energy, offering a reproducible framework for future NILM-enabled edge meter designs.

## 1. Introduction

The electrical energy consumption of consumer and commercial buildings accounts for 36% of the total electrical demand in the US and 25% in Europe [1,2], with an approximate increase of 3.4% per year [3]. In parallel, studies indicate that detailed analysis and real-time feedback on energy consumption can lead to up to 20% savings in energy consumption by detecting faulty devices and poor operational strategies [4]. Therefore, in the last few decades, extensive research in smart metering technologies, smart grid architectures, and data processing techniques has been carried out to reduce energy consumption [5]. To achieve this, accurate and fine-grained energy monitoring is needed [6], and energy disaggregation or Non-Intrusive Load Monitoring (NILM) has proven to be an essential technology for this [7].

NILM splits the energy consumption signal at the device level by measuring only the aggregated signal at the main inlet of a utility customer, i.e., with only one smart meter. The recorded aggregated energy measurements are typically transmitted via an IoT setup to a cloud server for further processing by an algorithm, like a deep machine learning pre-trained NILM model [8], to perform the energy disaggregation. Therefore, there is no need to connect a separate smart meter to each load to be monitored, since each of the target loads/appliances are monitored by the same single smart meter after AI-based processing of the recordings.

Common sampling rates of smart meters vary from one sample per second to one sample per ten minutes. The accuracy of NILM algorithms is proportional to the sampling frequency [9], due to the number of harmonics in the signature of each load that can be captured, however acquiring the aggregated signal at higher sampling frequencies would require a larger bandwidth to transmit the data to the cloud server and potentially more storage space within the cloud. Recent developments in edge AI computing [10] can be used to relocate the deep learning NILM to the edge, i.e., on the smart meter, to transmit only the detected load consumptions instead of the high sampling frequency measurements.

This article presents an instrumental prototype architecture of a smart meter with embedded capabilities for high-frequency NILM on the edge. The hardware implementation of the NILM smart meter is described and evaluated according to the analog measurement error of current and voltage signatures in the time and frequency domain. The proposed design combines a custom analog front-end and a real-time embedded AI pipeline, resulting in an edge-processing meter. The prototype architecture is evaluated for measurement fidelity, embedded runtime performance, and hardware efficiency across six microprocessor platforms. Additionally, we propose three new benchmarking metrics—accuracy-per-cost, accuracy-per-throughput, and accuracy-per-power—to guide hardware-software co-design in NILM-enabled smart meters, and to assess trade-offs and practical deployment considerations specifically in resource-constrained environments.

The remainder of the article is structured as follows: Section 2 provides an overview of the related works. In Section 3 the proposed architecture is presented. In Section 4 the performance is evaluated. Discussion and conclusions are provided in Section 5 and Section 6, respectively.

## 2. Related Works

The majority of the NILM approaches are software-based and have been evaluated mostly on publicly available datasets [7], while transfer learning approaches have been used in a few studies [11,12]. Only a few NILM approaches have been evaluated on hardware and tested using hardware implementations of smart meter architectures. Specifically, in [13], rule-based energy disaggregation was performed on a laboratory-based smart meter tested at a sampling frequency of 2 kHz. In [14], a smart meter and data acquisition method that characterizes load signatures based on the fingerprint of the root mean square (RMS) current, its average, and standard deviation was presented, which uses a dynamic window of samples based on the appliance type and a resolution of 24 bits at the analog–digital converter (ADC). In [15], time-frequency analysis was performed using signatures with 2D resolution based on short-time Fourier transform using a sampling frequency of 2 kHz and an ADC resolution of 12 bits. In [16], a real-time capable solution based on the Karhunen–Loeve expansion was used to perform disaggregation at low frequencies. The approach used a commercially available smart meter, the EKM-Omnimeter I v.3 [16], with a sampling frequency of 1 Hz and a measurement error of ±0.5%.

Some papers have introduced NILM-specific smart metering solutions. In [17], a low-cost smart meter was proposed that was based on an ARM Cortex-M4 processor architecture, while measurements were performed using split-core transformers. The meter had a WiFi interface for transmission of the measured data. The sampling frequency of the meter was 10 kHz, the delay for sending the data packages was 0.5 s, and the approximated cost was EUR 50. In [18], a proposed smart meter in the shape of a load plug was developed to use a voltage transformer and a hall effect current sensor. An Arduino Pro Mini with an external ESP8266 Wi-Fi module was used. The smart meter measured active power at a sampling frequency of 1 Hz. In [19], a Raspberry Pi-based smart metering solution for NILM was proposed to measure active and reactive power at a sampling frequency of 10 Hz, with a measurement error of approximately 3.5%, power consumption of 3.57 W, and approximate estimated cost in 2023 of EUR 50 for the materials and EUR 200 in total. In [20], a cost-effective embedded NILM smart meter was proposed that works with an internal sampling frequency of 10 kHz, 12-bit data resolution, and output processed values every 0.5 s. The measurement circuits used split coil transformers, while the data processing used a TI CC3200 MCU. In [21], a convolutional neural network (CNN) solution was proposed using an FPGA, with a sampling frequency of 4 kHz using a Zybo z7-20 board, and the acquired data were reshaped, forming 64×64 input windows. A data resolution of 3 bits was used in the CNN model.

In addition to NILM-specific metering solutions, general smart metering solutions have been proposed. An overview of smart meter technologies and specifically proposed metering solutions is provided in [22]. Another survey [23] focused on smart meters’ capabilities to detect energy fraud. In [24], a smart meter for general IoT applications in smart grids was proposed, working at a sampling frequency of up to 50 kHz and 24-bit resolution, and was based on an NI RIO 9626 board supported by a Xilinx LX45 FPGA architecture. The smart meter computed a set of 30 features, including electrical and statistical values suitable for NILM [25], and an error of less than 0.1% for current and voltage measurements was reported. In [26], an Arduino-based low-cost smart meter with a GSM module for data communication was proposed, achieving a measurement error of 0.8%. In [27], a secure energy smart meter was proposed that used encrypted data transfer and measured active and apparent power twice every 15 minutes.

## 3. Architecture

For a set of M−1 known loads/devices each consuming power $p_{m}\in R^{N}$, with 1≤m≤M−1 and *N* being the total number of samples within a time window, the aggregated power $p_{agg}\in R^{N}$ measured by the sensor will be:

(1) $$p_{agg}=f\left(p_{1},\cdots ,p_{M-1},e\right)=\sum_{m=1}^{M-1}p_{m}+e=\sum_{m=1}^{M}p_{m}$$

where $e=p_{M}\in R^{N}$ is the noise generated by one or more unknown devices (also referred to as ghost power [11]) and $f\left(\cdot \right)$ is the aggregation function. An NILM algorithm finds estimations ${\hat{p}}_{m},\hat{e}$ of the power consumption of each device *m* using an estimation method $f^{-1}\left(\cdot \right)$ with minimal estimation error and ${\hat{p}}_{M}=\hat{e}$, i.e.,

(2) $$\hat{P}=\left\{{\hat{p}}_{1},{\hat{p}}_{2},\cdots ,{\hat{p}}_{M-1},\hat{e}\right\}=f^{-1}\left(p_{agg}\right)$$

The hardware architecture and the software architecture are described in Section 3.1 and Section 3.2, respectively.

### 3.1. Hardware Architecture

**Figure 1:**
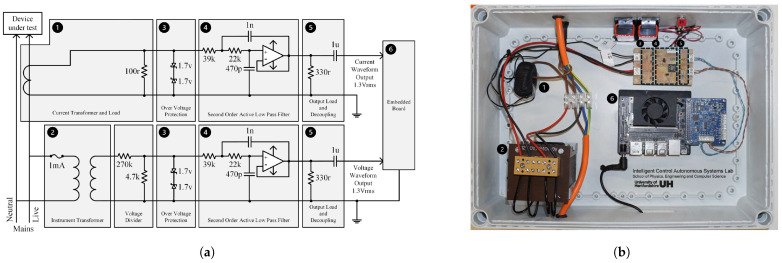
Hardware architecture and hardware implementation: (**a**) Architecture of the instrumental smart meter with embedded NILM including device under test (DUT), analog voltage, current measurement, and embedded boards. (**b**) Hardware implementation of the proposed smart meter. (1) Voltage measurement, (2) current measurement, (3) over-voltage protection, (4) low-pass filtering, (5) output decoupling, (6) embedded board.

The hardware architecture of the instrumental SM-NILM used in this work is presented in Figure 1a, and the hardware implementation of the smart meter in Figure 1b. The architecture comprises two parallel circuits, split into voltage and current waveform capturing and conditioning, which output the high-frequency voltage and current waveforms.

The voltage measurement circuit uses a custom-made instrument voltage transformer (ratio: 20:1, power rating: 0.01 VA, voltage factor: 1.2, compliant to IEC 61869-1&3 [28,29]) to isolate and reduce the mains supply (230 V) with a following voltage divider reducing the voltage level to 12 V and 1.3 V, respectively. The current measurement circuit uses a custom-made current transformer (ratio: 2000:1, power rating: 0.0085 VA, compliant to IEC 61869-1&2 [28,30]) in parallel to a resistor to transform the current consumed by the device under test (DUT) into a voltage waveform, with the voltage of the waveform being a linear conversion of the current. The resistor connected in parallel to the current transformer provides a maximum of 1.3 V at a nominal load of 3000 W, thus providing compatibility with audio-line levels.

Each circuit utilizes duplicated signal conditional sub-circuits, providing waveform amplitude limiting, anti-aliasing filtering, output load matching, and decoupling, following regular audio circuit compatibility. To bound the waveform’s amplitude, a pair of back-to-back Zener diodes is used in parallel to the output voltage signals (1.3 V) after the voltage divider and the current transformer, respectively. Therefore, the waveform amplitude is bounded at ±1.7 V to avoid damaging the following components. To select a suitable design point for the sampling frequency of the smart meter, the WHITE dataset [31] was used to investigate the frequency response of different devices.

**Figure 2:**
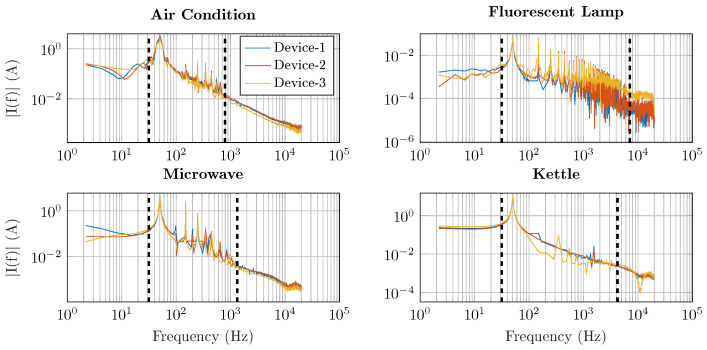
Current spectrum of four different devices from three manufacturers, each recorded at 44 kHz. Black dashed lines denote the relevant frequency content of each appliance.

As shown in Figure 2, each signal component appears in a specific frequency band, e.g., the AC at 30–700 Hz and the lamp at 30–7000 Hz. Visual investigation shows that the spectral content from all devices appears sufficiently up to 7 kHz, and thus a sampling frequency of 15 kHz was selected as the design point for the smart meter hardware to satisfy the Nyquist criterion and capture relevant frequency content from appliances. Consequently, an anti-aliasing filter is implemented using an active second-order low-pass filter, with a 6dB roll-off set to 7.5 kHz, thus allowing suitable high-frequency attenuation to mitigate aliasing for ADCs with a sampling rate of 15 kHz or greater. An operational amplifier (072 OPAMP) was used for each voltage and current measurement circuit. Finally, a decoupling capacitor was used to filter signals lower than 20 Hz (DC removal), thus ensuring 50 Hz mains voltage and current signals and higher frequency components could be passed to the ADC.

### 3.2. Software Architecture

**Figure 3:**
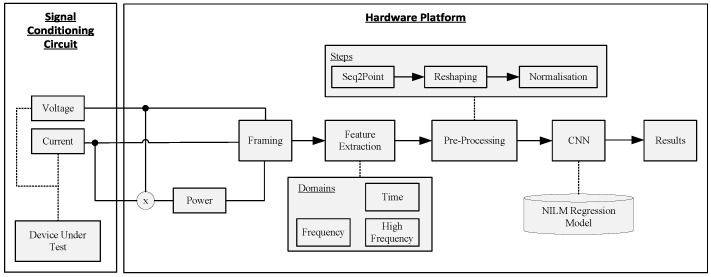
Software architecture including framing, feature extraction, pre-processing, and CNN regression.

The block diagram of the software architecture for the edge deployment is shown in Figure 3, showcasing the data flow from reading the raw electrical samples to executing the CNN model performing the disaggregation. A software application was developed to compare the performance of different hardware platforms, in terms of the accuracy of the models, the execution time of each stage (latency), and the overall throughput. The difference between the hardware setups lies in the CNN inference engine and the underlying technology for its acceleration. The rest of the data pipeline was kept the same as described below.

Each frame consists of 275 samples for the high-frequency domain, and 55 samples for the time and frequency domain using sub-sampling with a factor of five. Feature extraction is conducted on a frame basis, where various time domain and frequency domain features are derived from the signal. Although the same types of features are extracted across both frequency levels, the size of the FFT window used differs between the levels. Various pre-processing steps are applied to format the incoming data before sending it to the CNN. First, seq2point [32] is used to buffer ten consecutive frames into a single array using a sliding window approach with a step size of one sample. This technique takes the midpoint value of the target sequence using a sliding window of input data. This array is then reshaped into the appropriate single-point precision (FP32) format: Batch × Frame × Feature × Channels, where Batch and Channels are equal to one. Additionally, five different normalization techniques were explored (A)–(E) and are tabulated in Table 1, with (A) corresponding to ‘Without Normalization’.

**Figure 4:**
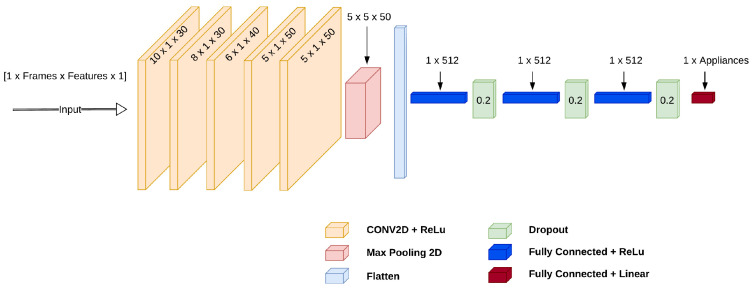
CNN architecture including five CNN and three DNN layers. The CNN layers are written in the format ‘conv(x,y)’ where *x* is the kernel size and *y* is the number of filters.

In Table 1, *x* is the raw input signal and $x^{\prime }$ is the pre-processed (normalized) input signal. Furthermore, $x_{min/max}$ is the minimum/maximum value, μ is the mean value, and σ is the standard deviation of the training data. The last module in the software architecture is the CNN model for NILM. The CNN architecture in [32] illustrated in Figure 4, using ten frames as input, was used for the regression step of NILM.

The architecture shown in Figure 4 was also used in [9] and in a similar form in [33,34], reporting competitive accuracy on high-frequency data for the UK-DALE dataset [35] and for low-frequency on the Ideal dataset [36].

### 3.3. Feature Extraction

The NILM features are split into two categories, namely the time and frequency domain, each consisting of nine features. Two sampling frequencies are considered. The features used in our evaluation are tabulated in Table 2.

Based on the features calculated in Table 2, the available electrical measurements of the smart meter, and the different normalization approaches, several evaluation scenarios were considered, and are listed in Table 3. Specifically, there are five feature domain scenarios, four measurement scenarios, and five normalization scenarios, resulting in 100 scenarios.

## 4. Performance Evaluation

The smart meter with embedded NILM architecture presented in Section 3 was evaluated in terms of the quality of the signal conditioning circuit implementation (Section 4.1), the NILM model accuracy (Section 4.2), and memory usage and runtime (Section 4.3).

### 4.1. Signal Conditioning Circuit

**Figure 5:**
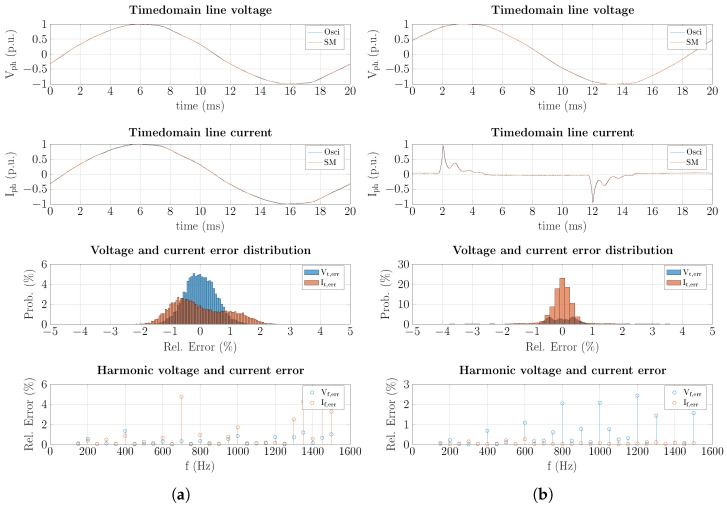
Comparison of waveforms of current and voltage measurements of one fundamental cycle for (**a**) DUT-1 (resistor kettle) and (**b**) DUT-2 (nonlinear fluorescent lamp) including error distribution for current and voltage waveforms as well as their harmonics.

The previously described experimental setup was implemented to evaluate the smart metering precision quality of the analog circuit. In this setup, a voltage probe was connected directly to the Live (L) and Neutral (N) wire, and a current probe clamp was attached to the Live supplying the DUT. The outputs of the voltage and current probes were connected to channels one (Ch1) and two (Ch2) of a high-speed oscilloscope (LeCroy SDA 760Zi-A). The voltage and current outputs from the smart meter hardware were then connected to channels three (Ch3) and four (Ch4) of the same oscilloscopes, allowing a direct comparison of the waveforms in the time domain. Specifically, two different DUTs were evaluated. The first one (DUT-1) was a purely resistive load (kettle), and the second one (DUT-2) was a highly nonlinear load (fluorescent lamp). The output waveform of current and voltage, as well as the distribution of the residual signals, are illustrated for one fundamental cycle in Figure 5.

As shown in Figure 5, the voltage and current waveforms of both DUTs are measured with a maximum error of less than ±2%. The error was averaged over ten electrical periods for the two DUTs. The measurement error results for current and voltage signals are tabulated in Table 4.

As tabulated in Table 4, voltage measurement errors are below 0.5% and the current MAE is below 1.0%, while only the current RMSE exceeds 1.0% in the case of the DUT-2.

### 4.2. NILM Modeling

To evaluate the performance and training of the NILM models, the REDD [37] dataset was employed, as the most widely used NILM dataset containing low- and high-frequency data. The REDD dataset is described in Table 5.

The high-frequency data of house three of REDD were used, and the performance was evaluated in terms of estimation accuracy as described in (3):

(3) $$E_{ACC}=1-\frac{\sum_{t=1}^{T}\sum_{m=1}^{M}\left|{\hat{p}}_{m}^{t}-p_{m}^{t}\right|}{2\sum_{t=1}^{T}\sum_{m=1}^{M}\left|p_{m}^{t}\right|}$$

where ${\hat{p}}_{m}^{t}$ and $p_{m}^{t}$ are the predicted and the ground-truth power consumption for the *m*-th device and the *t*-th frame. The NILM performance was evaluated for the experimental protocols described in Section 3.3, and the results are tabulated in Table 6.

As shown in Table 6, voltage-only based features (#1, 5, 9, 13, 17) have the lowest accuracy values across all normalization approaches (A–E), as voltage in residential settings is typically regulated by the utility and remains relatively constant, thus offering minimal discriminative information on appliance-level energy usage. Consequently, voltage-only feature sets were excluded from the further analyses in the remainder of the study. Among current-based features, TF-I (#14) achieved the highest accuracy at 83.83% (under normalization scheme C), suggesting that time-frequency domain features extracted from current signals are particularly informative for appliance disaggregation. Similarly, HF-I (#10) and F-I (#6) also showed robust performance, with accuracies of 82.67% and 81.97%, respectively. These results show that current signal variations provide a rich source of information to distinguish appliance signatures, especially when enhanced through frequency-domain analysis or time-frequency representations. In the power-based features, T-P (#3) achieved the highest overall accuracy of 85.54%, under the normalization scheme C. This suggests that temporal patterns of power consumption are particularly distinctive and can effectively capture the operating states of various appliances. Furthermore, other power-based features such as TF-P (#15) and TFHF-P (#19) also demonstrated high accuracies (above 83%), showing the importance of the power signal in energy disaggregation. When combining voltage, current, and power features, configurations like T-VIP (#4) and TF-VIP (#16) reached accuracies of 85.09% and 85.94%, respectively, under normalizations C and A. These results indicate that multi-modal feature fusion can further enhance NILM performance, as it enables the model to combine complementary information across electrical domains.

The next step was to optimize the best-performing model (#3) using state-of-the-art acceleration frameworks, namely the TF-Lite, eIQ, OpenVINO, TensorRT, and Vitis-AI. Table 7 tabulates the NILM accuracy of the best model (#3) for different acceleration frameworks and the corresponding difference from the baseline framework without acceleration (TensorFlow). The average acceleration improvement across all NILM models using Z-score normalization without voltage-only features is also tabulated in Table 7.

When models were quantized for INT8 deployment without normalization, significant drops in $E_{ACC}$ were observed, with models losing up to 50% accuracy. Upon investigating the data, it was found that overflows occurred when the activations and weights were converted to INT8, which caused these substantial accuracy drops. This was not observed with models using normalization (B, C, D, E). For the normalization using C (Z-score), when quantizing to FP16, there was no loss in NILM accuracy. However, for INT8 quantization, there was a noticeable drop in $E_{ACC}$, with some hardware acceleration frameworks (TFLITE-PTQ INT8, TensorRT INT8, Vitis-AI PTQ INT8 and QAT INT8) presenting a loss of up to 6.0%. The exception was eIQ PTQ-INT8, where quantization of the model reduced the accuracy by 47.5%, while the TF-LITE had a loss of up to 7.7%. Notably, QAT consistently outperformed PTQ approaches, demonstrating improved resilience in quantization-induced distortions. When using FP16 quantization, the original FP32 performance was preserved across all frameworks, including TF-Lite, TensorRT, and OpenVINO.

### 4.3. NILM on Edge Hardware

Six hardware boards were evaluated for the NILM edge implementation: the Raspberry Pi 4 (RP4), Intel Neural Compute Stick 2 (NCS2), NXP i.MX 8M Plus (IMX8), NVIDIA Jetson Nano (NANO), Jetson Xavier NX (XAVIERNX), and lastly the Ultra96v1 (ULTRA96V1) AMD-Xilinx MPSoC FPGA. The core features of the evaluated boards are tabulated in Table 8, showing their differences in processors, memory, deep learning acceleration, power consumption, and cost.

An end-to-end NILM on the edge evaluation was performed using the above boards. Every benchmark was executed for 30 s to average the results and remove outliers. Five metrics of performance were considered, namely the power consumption (W), feature extraction latency (ms), NILM model latency (ms), energy efficiency (FPS/W), and cost efficiency (FPS/$). In the context of this article, frames per second (FPS) is the number of frames of energy data that can be processed in one second.

**Figure 6:**
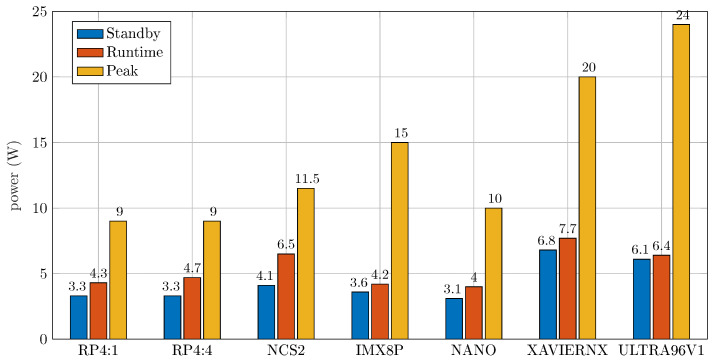
Average power consumption (standby, runtime, and peak operation) for different boards. RP4 utilizes one thread (RP4:1) and four threads (RP4:4).

**(1) Power Consumption:** To measure the power consumption, a power meter was used to calculate the standby, the runtime, and the peak power consumption. The average power consumption of the different NILM models for the evaluated hardware boards is shown in Figure 6. The board with the lowest runtime power consumption was NANO, while XAVIERNX had the highest power consumption.

**(2) Feature Extraction Latency: **Table 9 tabulates the latency of feature extraction for one frame on the CPU of each board. Voltage/current/power measurements had the same execution times. For the time- and frequency-domain, RP4 (64-bit OS) offered the lowest latency, due to the highest CPU frequency and the wide SIMD instruction set, enabling more operations per second, and is only outperformed by XAVIERNX in the high-frequency domain. Lastly, the slowest board was ULTRA96V1 in all cases, as it had the lowest CPU frequency.

**(3) Model Latency:** The execution times of the best-performing model (#3) for each hardware accelerator, except RP4 which does not support AI acceleration, are shown in Table 10. The five smallest models in terms of number of parameters (#2, 3, 4, 6, 7) have the lowest latency on IMX8P, while XAVIERNX achieved the best latency results across all models. The slowest performing device is the RP4 when using a single thread (RP4:1), except for the three smallest models (#2, 3, 4), where NCS2 is the slowest board. The XAVIERNX board has the smallest variation in execution time (0.57–0.91 ms) across all tested models, followed by IMX8P (0.49–2.82 ms), and NANO (0.98–3.63 ms), while the largest variation was on RP4:1 (1.19–45.37 ms).

**Figure 7:**
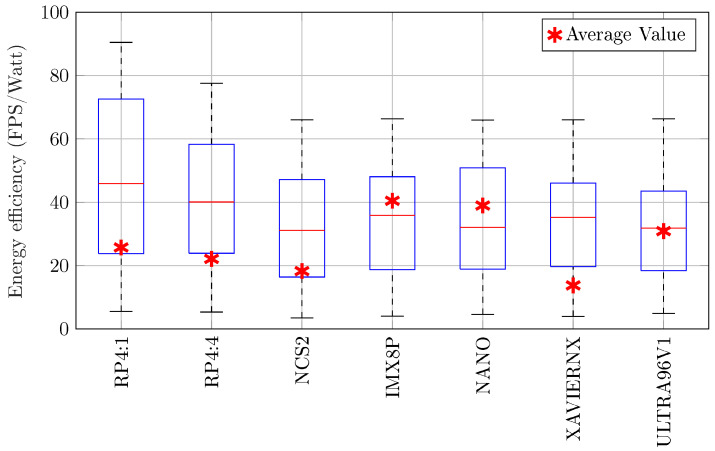
Energy efficiency (FPS/watt) of the boards, including minimum, maximum, median, and average values.

**(4) Energy Efficiency:** To benchmark the energy efficiency of each hardware board, throughput per watt was evaluated. Throughput included the end-to-end NILM data pipeline, the pre-processing, and the NILM model inference. Figure 7 shows the efficiency results for each board. On average, the most efficient across all models is the IMX8P with 40.5 FPS/watt, while the least efficient is the XAVIERNX with 13.8 FPS/watt.

**Figure 8:**
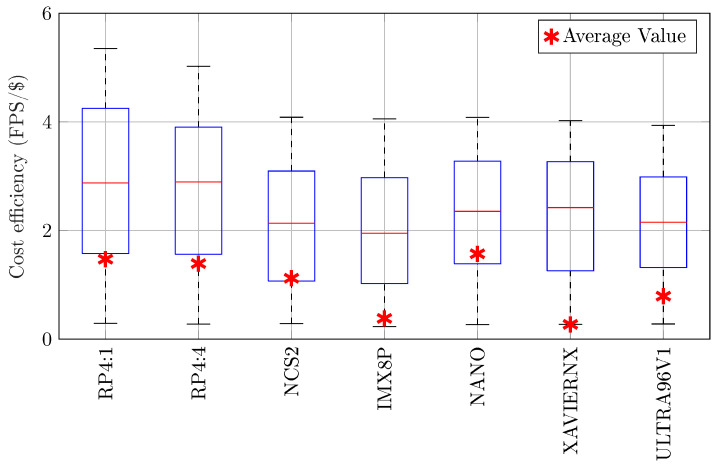
Cost efficiency (FPS/$) of the evaluated boards, including minimum, maximum, median, and average values.

**(5) Cost Efficiency:** Similarly, the cost efficiency in terms of throughput per cost was evaluated, and the results are illustrated in Figure 8. On average, NANO was the best in cost efficiency with 1.57 FPS/$, while the worst board was XAVIERNX with 0.27 FPS/$.

## 5. Discussion

Further to the performance evaluation of the prototype smart meter presented in Section 4, the measurement error during transient events is investigated in Section 5.1, new NILM-on-Hardware metrics are introduced in Section 5.2, and analysis on the trade-off between NILM models’ size and accuracy is provided in Section 5.3.

### 5.1. Measurements During Transient Events

**Figure 9:**
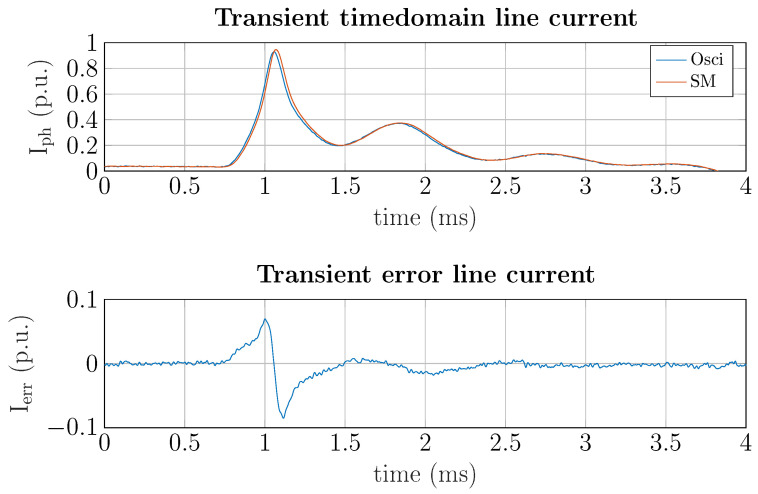
Transient current waveform of DUT-2 (fluorescent lamp) for an on/off switching event.

In addition to disaggregating the household energy consumption, some approaches focus on identifying device operation [38]. Therefore, the transient switching instances between on/off operations are used in feature extraction and evaluation of appliance activity in the time, frequency, or time–frequency domain. To accurately compute these features, measuring with precision during transient operations is crucial. Therefore, the transient behavior of the non-linear DUT-2 (fluorescent lamp) is investigated as it has the largest rate of change in current ($\frac{dI_{ph}}{dt}$). A switching event is illustrated in Figure 9.

As shown in Figure 9, even for transient events shorter than 1 ms, the maximum error in the current measurement is well below ±10%, while the average error over the transient period is approximately 1.1%. Statistical current features during transient events were calculated using the smart meter and oscilloscope measurements for reference. The relative and absolute errors for some of the most widely-used statistical features are tabulated in Table 11.

### 5.2. NILM-on-Hardware Performance Metrics

Three new performance metrics are introduced to evaluate the NILM performance for the utilized hardware. First, the $E_{ACC}$ per energy metric ($E_{ACC}$/Watt) normalizes the energy disaggregation accuracy to the energy consumption of the hardware. Second, the $E_{ACC}$ per cost metric ($E_{ACC}$/$) normalizes the energy disaggregation accuracy to the cost of the hardware. Third, the $E_{ACC}$ per FPS metric ($E_{ACC}$/FPS) estimates the energy disaggregation accuracy per hardware throughput (FPS) and assesses the accuracy loss due to the hardware deployment. The three metrics consider the hardware limitations affecting NILM performance when implemented on the edge (on a smart meter device). The results for all evaluated boards are tabulated in Table 12.

As shown in Table 12 the NANO is the best-performing board concerning energy-efficient NILM, achieving 20.6% NILM accuracy per watt. However, the most cost-efficient NILM board is the Raspberry Pi (RP4) achieving 1.07% accuracy per $ of hardware cost. The best NILM accuracy was achieved by XAVIERNX with 1.46% NILM accuracy per FPS.

### 5.3. Model Size and Accuracy

**Figure 10:**
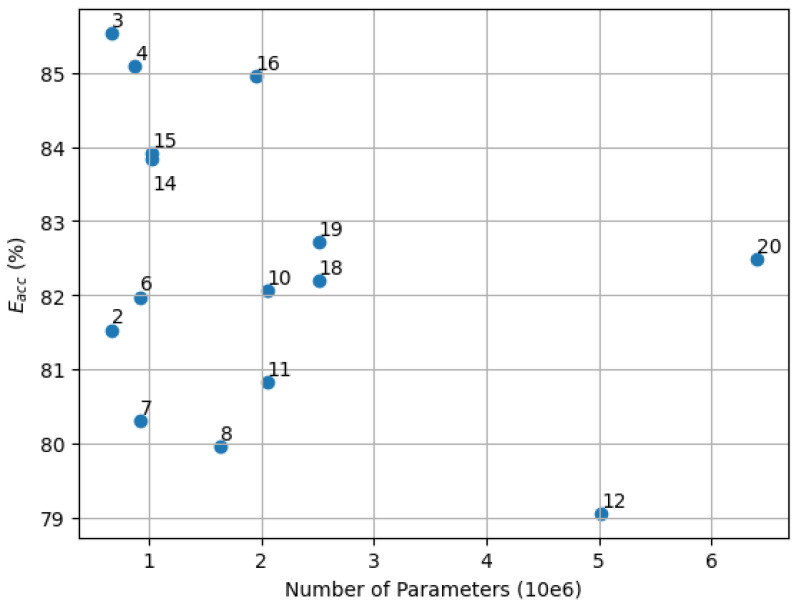
Relation of the NILM model size to the NILM accuracy for different feature and domain setups. Each dot represents a setup as defined in Table 6.

Since the memory requirements and the runtime are mostly determined by the number of the parameters of each NILM model, the relationship of the NILM model size to the NILM accuracy was investigated for the setups of Table 6 using z-score normalization. The results are illustrated in Figure 10.

As shown in Figure 10 model #3 (time domain and power) provides the best trade-off between the number of model parameters and NILM accuracy achieving energy disaggregation accuracy of 85.5% using $6.7\times 10^{5}$ model parameters.

## 6. Conclusions

The architecture of a smart meter prototype for high sampling frequency and energy disaggregation on the edge was presented. The smart meter prototype consists of a custom signal conditioning circuit interconnected to an embedded board performing energy disaggregation using a deep-learning model. Different feature setups and sampling frequencies were evaluated with respect to the accuracy of the energy disaggregation models and the edge device power consumption, throughput, and latency across different hardware platforms. In addition, three new metrics were introduced to assess the performance of NILM on edge hardware.

The evaluation of the smart meter prototype showed state-of-the-art accuracy in the analog measurements, including measurements at transient periods of devices. Moreover, the achieved energy disaggregation performances of the hardware-accelerated NILM models indicated the ability to execute NILM algorithms on the edge, instead of transmitting the measurements to the cloud, which would be prohibitive for high sampling frequencies. We deem the presented architecture to be used for instrumentation purposes, and in further development of smart meters with NILM on-edge capabilities.

## Figures and Tables

**Table 1:** Normalization methods (Norm) for input features.

Norm	Amplitude Range	Equation
A	Without Normalization	$x^{\prime }=x$
B	[0, 1]	$x^{\prime }=\frac{x-x_{min}}{x_{max}-x_{min}}$
C	Z-Score	$x^{\prime }=\frac{x-\mu }{\sigma }$
D	[−128, 127]	$x^{\prime }=\frac{x-x_{min}}{x_{max}-x_{min}}\times 255-128$
E	[0, 255]	$x^{\prime }=\frac{x-x_{min}}{x_{max}-x_{min}}\times 255$

**Table 2:** Time and frequency domain features.

Time ID	Time Name	Freq. ID	Freq. Name
T-1	Minimum	F-1	Spectral Amplitude
T-2	Maximum	F-2	Spectral Centroid
T-3	Variance	F-3	Spectral Spread
T-4	Standard Deviation	F-4	Spectral Skewness
T-5	25% Percentile	F-5	Spectral Kurtosis
T-6	50% Percentile	F-6	Spectral Entropy
T-7	75% Percentile	F-7	Spectral Flatness
T-8	Kurtosis	F-8	Spectral Crest
T-9	Skewness	F-9	Spectral Slope

**Table 3:** Abbreviations for different experimental protocols, including feature domain, measurement, and normalization.

Abv. Feature	Name Feature	Abv. Meas.	Name Meas.	Abv. Norm.	Name Norm.
T	Time	V	Voltage	A	None
F	Frequency	I	Current	B	[0, 1]
HF	High Frequency	P	Power	C	Z-Score
TF	Time + F	VIP	All	D	[−128, 127]
TFHF	Time + F + HF	-	-	E	[0, 255]

**Table 4:** Averaged measurement errors (in percentages) of voltage and current waveforms for DUT-1 (resistor kettle) and DUT-2 (nonlinear fluorescence lamp).

DUT	Current Waveform		Voltage Waveform	
	MAE	RMSE	MAE	RMSE
1	0.71%	0.85%	0.43%	0.53%
2	0.49%	1.17%	0.34%	0.39%

**Table 5:** Overview of the REDD dataset.

Houses	Year	Country	Devices	Feature	Sampling
1	2011	US	15	P	1/3 Hz
2			9	P	1/3 Hz
3			19	V, I, P	15 kHz
4			17	P	1/3 Hz
5			22	P	1/3 Hz
6			14	V, I, P	15 kHz

**Table 6:** NILM accuracy ($E_{ACC}$) for different feature setups and normalization approaches (A–E).

Setup	Features	A	B	C	D	E
1	T-V	64.84%	64.79%	57.03%	64.84%	64.79%
2	T-I	82.81%	64.80%	81.53%	79.91%	82.69%
3	T-P	84.23%	64.79%	**85.54%**	65.22%	82.61%
4	T-VIP	85.65%	80.59%	85.09%	68.07%	82.60%
5	F-V	64.75%	64.78%	63.15%	64.80%	64.77%
6	F-I	82.08%	77.46%	**81.97%**	78.93%	81.81%
7	F-P	81.85%	77.51%	80.30%	76.47%	80.41%
8	F-VIP	81.84%	78.24%	79.96%	80.93%	81.87%
9	HF-V	64.78%	64.79%	61.95%	64.80%	64.80%
10	HF-I	**82.67%**	81.64%	82.06%	79.23%	81.54%
11	HF-P	80.22%	81.11%	80.82%	78.72%	78.37%
12	HF-VIP	81.60%	77.77%	79.05%	82.53%	77.30%
13	TF-V	64.79%	64.84%	61.21%	64.80%	64.80%
14	TF-I	80.50%	81.91%	83.83%	83.06%	84.58%
15	TF-P	83.03%	79.40%	83.91%	78.19%	82.95%
16	TF-VIP	**85.94%**	81.47%	84.96%	72.32%	82.79%
17	TFHF-V	64.80%	59.06	60.60%	64.84%	56.12%
18	TFHF-I	83.00%	64.82	82.19%	77.89%	82.65%
19	TFHF-P	83.30%	81.69	82.72%	80.44%	82.14%
20	TFHF-VIP	**83.93%**	83.59	82.48%	65.06%	83.77%

**Table 7:** Accuracy of optimized NILM models for different hardware acceleration frameworks and data types.

		T-P (#3)		Average	
Framework	Data Type	$E_{\mathrm{ACC}}$	Delta	$E_{\mathrm{ACC}}$	Delta
TensorFlow	FP32	85.54%	-	82.43%	-
TF-Lite	FP16	85.54%	0.00%	82.42%	0.01%
	DINT8	85.76%	−0.22%	82.63%	−0.20%
	PTQ INT8	77.83%	7.71%	70.55%	11.88%
	QAT INT8	84.12%	1.42%	80.43%	2.00%
eIQ	FP16	85.54%	0.00%	82.42%	0.01%
	PTQ INT8	38.06%	47.48%	55.54%	26.88%
OpenVINO	FP16	85.54%	0.00%	82.42%	0.01%
TensorRT	FP16	85.54%	0.00%	82.42%	0.01%
	INT8	83.85%	1.69%	80.59%	1.84%
Vitis-AI	PTQ INT8	76.26%	9.28%	76.42%	6.00%
	QAT INT8	80.76%	4.78 %	77.85%	4.57%

**Table 8:** Hardware boards evaluated and their core features. TDP stands for thermal design power (in watts).

HW Board	CPU	Memory	Accelerator	TDP (W)	Cost ($)
RP4	Cortex-A72	8 GB	-	9	75
NCS2 (+RP4)	N/A	500 MB	VPU	2.5 (9)	99 (75)
IMX8P	Cortex-A53	6 GB	NPU	15	449
NANO	Cortex-A57	4 GB	128 CUDA	10	99
XAVIERNX	Carmel v8.2	8 GB	384 CUDA	20	399
ULTRA96V1	Cortex-A53	2 GB	B2304 DPU	24	249

**Table 9:** Feature extraction latency (ms) for different hardware platforms in the time- and frequency-domain.

Hardware	Time	Low-Frequency	High-Frequency
RP4_64bit	0.074	0.831	3.092
RP4_32bit	0.087	0.990	3.682
IMX8P	0.182	1.530	5.740
NANO	0.127	1.456	5.406
XAVIERNX	0.098	0.839	2.949
ULTRA96V1	0.186	1.780	6.644

**Table 10:** NILM model execution time (ms) for different hardware boards and features.

Setup	Parameters	Features	RP4:1	RP4:4	NCS2	IMX8P	NANO	XAVIERNX	ULTRA96V1
2	671 k	T-I	1.19	0.74	2.51	0.50	0.98	0.62	1.05
3	671 k	T-P	1.19	0.75	2.47	0.49	1.00	0.63	1.05
4	876 k	T-VIP	2.55	1.19	2.57	0.55	0.99	0.65	1.20
6	927 k	F-I	3.01	2.81	2.56	0.56	1.14	0.64	1.23
7	927 k	F-P	3.00	2.91	2.61	0.55	1.05	0.62	1.23
8	1.6 m	F-VIP	7.80	3.07	3.29	0.80	1.31	0.67	1.80
10	2.0 m	HF-I	10.58	4.39	3.60	0.89	1.50	0.63	2.15
11	2.0 m	HF-P	10.54	4.09	3.47	0.92	1.51	0.63	2.15
12	5.0 m	HF-VIP	35.09	13.43	7.48	2.32	2.93	0.81	4.84
14	1.0 m	TF-I	3.68	1.76	2.64	0.61	0.98	0.57	1.34
15	1.0 m	TF-P	3.66	1.60	2.62	0.62	0.99	0.58	1.33
16	1.9 m	TF-VIP	9.71	3.81	3.41	0.86	1.45	0.64	2.05
18	2.5 m	TFHF-I	13.97	5.54	4.03	1.02	1.69	0.68	2.48
19	2.5 m	TFHF-P	13.93	5.70	4.06	1.05	1.69	0.71	2.49
20	6.4 m	TFHF-VIP	45.37	18.92	8.76	2.82	3.63	0.91	6.14

**Table 11:** Relative (%) and absolute error for statistical features during a transient event for a non-linear load. ‘Max’, ‘Min’, ‘Avg’, ‘Rms’, ‘Std’, and ‘En’ denote the signal’s maximum, minimum, average, root-mean-square, standard deviation, and energy value.

Error	Max	Min	Avg	Rms	Std	En
Relative (%)	1.91	0.56	1.87	1.76	1.71	1.38
Absolute	1.78	0.26	0.23	0.38	0.30	1.03

**Table 12:** Proposed NILM-on-Hardware performance for the different evaluated boards using the best-performing feature setup T-P (#3).

Board	$E_{\mathrm{ACC}}/\mathrm{Watt}$	$E_{\mathrm{ACC}}/\$$	$E_{\mathrm{ACC}}/\mathrm{FPS}$
RP4:1	18.7	**1.07**	1.39
RP4:4	17.1	**1.07**	1.44
NCS2	12.7	0.78	1.30
IMX8P	16.8	0.16	0.86
NANO	**20.1**	0.83	1.01
XAVIERNX	10.5	0.20	**1.46**
ULTRA96V1	12.6	0.32	0.79

## Data Availability

The original contributions presented in this study are included in the article. Further inquiries can be directed to the corresponding author.

## References

[1] Elma O., Selamoğullar U.S. A survey of a residential load profile for demand side management systems. Proceedings of the 2017 IEEE International Conference on Smart Energy Grid Engineering (SEGE).

[2] Eurostat (2018). Energy Statistics—An Overview. https://ec.europa.eu/eurostat/statistics-explained/index.php?title=Energy_statistics_-_an_overview.

[3] Yu B., Tian Y., Zhang J. (2015). A dynamic active energy demand management system for evaluating the effect of policy scheme on household energy consumption behavior. Energy.

[4] He D., Lin W., Liu N., Harley R.G., Habetler T.G. (2013). Incorporating non-intrusive load monitoring into building level demand response. IEEE Trans. Smart Grid.

[5] Çimen H., Çetinkaya N., Vasquez J.C., Guerrero J.M. (2021). A Microgrid Energy Management System Based on Non-Intrusive Load Monitoring via Multitask Learning. IEEE Trans. Smart Grid.

[6] Chiş A., Rajasekharan J., Lunden J., Koivunen V. Demand response for renewable energy integration and load balancing in smart grid communities. Proceedings of the 2016 24th European Signal Processing Conference (EUSIPCO).

[7] Schirmer P.A., Mporas I. (2021). On the non-intrusive extraction of residents’ privacy-and security-sensitive information from energy smart meters. Neural Comput. Appl..

[8] Wang L., Mao S., Wilamowski B.M., Nelms R.M. (2021). Pre-trained models for non-intrusive appliance load monitoring. IEEE Trans. Green Commun. Netw..

[9] Schirmer P.A., Mporas I. (2021). Double Fourier integral analysis based convolutional neural network regression for high-frequency energy disaggregation. IEEE Trans. Emerg. Top. Comput. Intell..

[10] Hu H., Tang L. (2020). Edge intelligence for real-time data analytics in an IoT-based smart metering system. IEEE Netw..

[11] Schirmer P.A., Mporas I. (2023). Non-Intrusive Load Monitoring: A Review. IEEE Trans. Smart Grid.

[12] Schirmer P.A., Mporas I. (2022). Device and Time Invariant Features for Transferable Non-Intrusive Load Monitoring. IEEE Open Access J. Power Energy.

[13] Bilski P., Winiecki W. The rule-based method for the non-intrusive electrical appliances identification. Proceedings of the IDAACS’2015: Proceedings of the 2015 IEEE 8th International Conference on Intelligent Data Acquisition and Advanced Computing Systems: Technology and Applications (IDAACS).

[14] Tran T.T., Lee G.D., Pham T.X., Kim G.J., Van Dang C., Kim J.W., Kang B. Identification of in-home appliances through analysis of current consumption. Proceedings of the 10th International Conference on Ubiquitous Information Management and Communication.

[15] Lin Y.H., Tsai M.S. (2014). Development of an Improved Time–Frequency Analysis-Based Nonintrusive Load Monitor for Load Demand Identification. IEEE Trans. Instrum. Meas..

[16] Welikala S., Thelasingha N., Akram M., Ekanayake P.B., Godaliyadda R.I., Ekanayake J.B. (2019). Implementation of a robust real-time non-intrusive load monitoring solution. Appl. Energy.

[17] Nardello M., Rossi M., Brunelli D. A low-cost smart sensor for non intrusive load monitoring applications. Proceedings of the 2017 IEEE 26th International Symposium on Industrial Electronics (ISIE).

[18] Aftab M., Chau C.K., Khonji M. Real-time appliance identification using smart plugs: Demo abstract. Proceedings of the Eighth International Conference on Future Energy Systems.

[19] Winkler J., Bousbiat H., Jost S., Elmenreich W. Energy Disaggregation with NILM on a Raspberry Pi with Smart-Metering Extension. Proceedings of the 2023 2nd International Conference on Power Systems and Electrical Technology (PSET).

[20] Nardello M., Rossi M., Brunelli D. An innovative cost-effective smart meter with embedded non intrusive load monitoring. Proceedings of the 2017 IEEE PES Innovative Smart Grid Technologies Conference Europe (ISGT-Europe).

[21] Tapiador M., de Diego-Otón L., Hernández Á., Nieto R. Implementing a CNN in FPGA Programmable Logic for NILM Application. Proceedings of the 2023 38th Conference on Design of Circuits and Integrated Systems (DCIS).

[22] Orlando M., Estebsari A., Pons E., Pau M., Quer S., Poncino M., Bottaccioli L., Patti E. (2021). A smart meter infrastructure for smart grid IoT applications. IEEE Internet Things J..

[23] Xia X., Xiao Y., Liang W., Cui J. (2022). Detection methods in smart meters for electricity thefts: A survey. Proc. IEEE.

[24] Morello R., De Capua C., Fulco G., Mukhopadhyay S.C. (2017). A smart power meter to monitor energy flow in smart grids: The role of advanced sensing and IoT in the electric grid of the future. IEEE Sens. J..

[25] Schirmer P.A., Mporas I. (2019). Statistical and electrical features evaluation for electrical appliances energy disaggregation. Sustainability.

[26] Patel H.K., Mody T., Goyal A. Arduino based smart energy meter using GSM. Proceedings of the 2019 4th International Conference on Internet of Things: Smart Innovation and Usages (IoT-SIU).

[27] Hseiki H., El-Hajj A., Ajra Y., Hija F., Haidar A. (2024). A secure and resilient smart energy meter. IEEE Access.

[28] (2023). Instrument Transformers—Part 1: General Requirements.

[29] (2011). Instrument Transformers—Part 3: Additional Requirements for Inductive Voltage Transformers.

[30] (2012). Instrument Transformers—Part 2: Additional Requirements for Current Transformers.

[31] Kahl M., Haq A.U., Kriechbaumer T., Jacobsen H.A. WHITED-A Worldwide Household and Industry Transient Energy Data Set. Proceedings of the 3rd International Workshop on Non-Intrusive Load Monitoring.

[32] Zhang C., Zhong M., Wang Z., Goddard N., Sutton C. Sequence-to-point learning with neural networks for non-intrusive load monitoring. Proceedings of the AAAI Conference on Artificial Intelligence.

[33] Wu Q., Wang F. (2019). Concatenate Convolutional Neural Networks for Non-Intrusive Load Monitoring across Complex Background. Energies.

[34] Brewitt C., Goddard N. (2018). Non-intrusive load monitoring with fully convolutional networks. arXiv.

[35] Kelly J., Knottenbelt W. (2015). The UK-DALE dataset, domestic appliance-level electricity demand and whole-house demand from five UK homes. Sci. Data.

[36] Pullinger M., Kilgour J., Goddard N., Berliner N., Webb L., Dzikovska M., Lovell H., Mann J., Sutton C., Webb J. (2021). The IDEAL household energy dataset, electricity, gas, contextual sensor data and survey data for 255 UK homes. Sci. Data.

[37] Kolter J.Z., Johnson M.J. REDD: A public data set for energy disaggregation research. Proceedings of the Workshop on Data Mining Applications in Sustainability (SIGKDD).

[38] Le T.-T.-H., Heo S., Kim H. (2021). Toward Load Identification Based on the Hilbert Transform and Sequence to Sequence Long Short-Term Memory. IEEE Trans. Smart Grid.

